# Dr. Kadambini Ganguly (1861-1923): A Pioneer in Indian Medicine

**DOI:** 10.7759/cureus.63543

**Published:** 2024-06-30

**Authors:** Sheuli Paul, Shradha Salunkhe, Shailaja V Mane, Poulomi A Ghosh

**Affiliations:** 1 Paediatrics, Dr. D. Y. Patil Medical College, Hospital and Research Centre, Pune, IND; 2 Psychiatry, Dr. D. Y. Patil Medical College, Hospital and Research Centre, Pune, IND

**Keywords:** kadambini ganguly, social reform, historical vignettes, pioneering women, gender equality, women's rights, female physicians, medical education, women's healthcare, indian medicine

## Abstract

Dr. Kadambini Ganguly was a trailblazing Indian physician and social reformer. As one of the first female graduates and practitioners of Western medicine in India, she broke numerous barriers in a field dominated by men. Her contribution to medicine, particularly in women's healthcare, and her engagement in social reform through the Brahmo Samaj and the Indian National Congress, caused significant progress toward gender equality and social justice. This article looks back on her academic accomplishments, medical career, social activism, and lasting legacy, emphasizing her profound influence on medicine and society in India.

## Introduction and background

**Figure 1:**
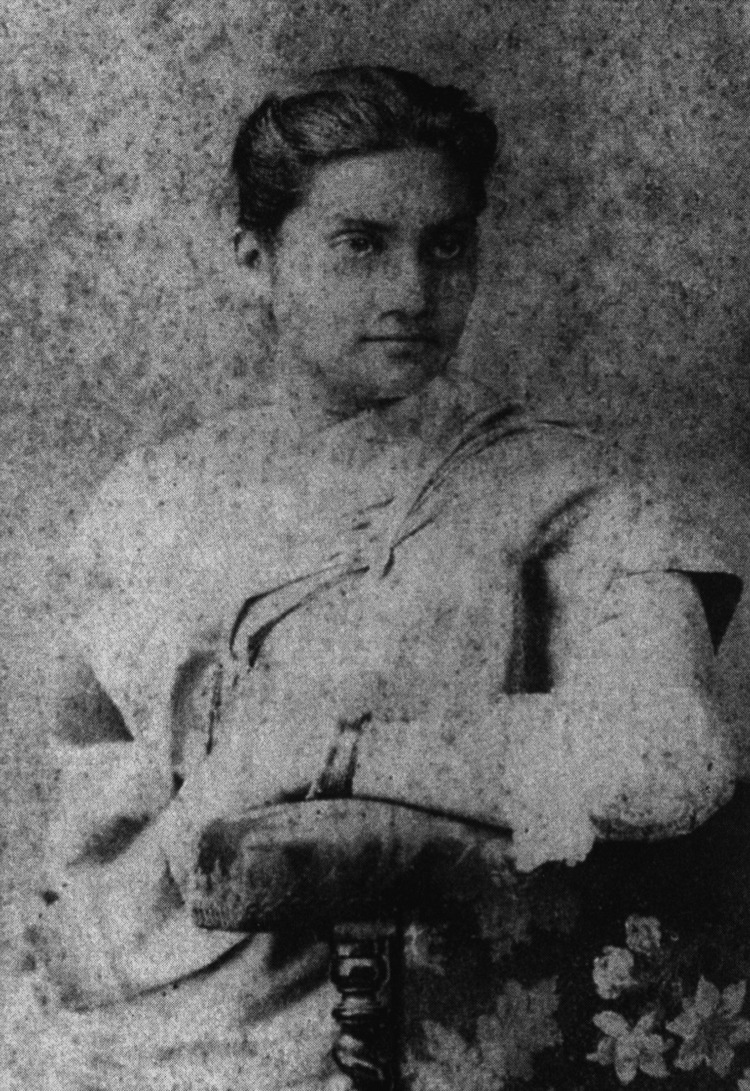
Dr. Kadambini Ganguly Permission obtained from India International Centre Quarterly [1].

Dr. Kadambini Ganguly (née Basu) was born on July 18, 1861, in Bhagalpur, Bihar, British India, to Bengali parents (Figure 1). Her father, Brajakishore Basu, who belonged to the Barishal district of Bengal Presidency (currently Bangladesh), was an influential Brahmo reformer actively involved in an organization working for the emancipation of women. The progressive values he instilled in his daughter shaped much of Kadambini’s upbringing. The Brahmo Samaj, founded by Rammohan Roy, was a progressive social reform movement within Hinduism and heavily influenced Kadambini Ganguly's early life and development. This environment, which championed the values of education and gender equality, contributed significantly to shaping her exemplary career and lifelong dedication to social justice.

Kadambini's academic path was extraordinary for her era, marked by pioneering accomplishments. She enrolled at Bethune College, associated with the University of Calcutta, where she obtained her Bachelor of Arts degree in 1883. She and her contemporary Chandramukhi Basu were the first female graduates in India and the British Empire [2]. Undeterred by the barriers she faced as a woman in a male-dominated society, Kadambini pursued her medical studies at the Calcutta Medical College, encouraged by her husband Dwarakanath Ganguly, and supported by social reformers like Ishwar Chandra Vidyasagar. Given the societal norms and resistance she encountered, this was an extraordinary achievement, as Calcutta Medical College changed its age-old tradition of registering only male students for studying medicine [3].

In 1886, she was awarded the Graduate of Bengal Medical College (GBMC) degree from Calcutta Medical College [4]. The same year, Anandibai Joshi was awarded a degree in medicine by Drexel University, USA, but could not practice medicine as she passed away in 1887 due to tuberculosis. Dr. Ganguly is regarded as the first female physician to practice Western medicine in India (Figure 1).

## Review

Medical career and achievements

Dr. Ganguly's medical career was truly remarkable, emphasizing the significance of her academic achievements. A word about Ganguly reached Florence Nightingale, and she wrote to a friend, “Do you know or could tell me anything about Mrs. Ganguly, or give me any advice? She has already passed what is called the first licentiate in medicine and surgery examinations and is to go up for the final examination in March next. This young lady, Mrs. Ganguly, married after she made up her mind to become a doctor and has had one if not two children since. But she was absent only thirteen days for her lying-in and did not miss, I believe, a single lecture!” [5].

In 1888, Dr. Ganguly was appointed as an assistant to the Lady Dufferin Hospital, Calcutta, which was dedicated to women's healthcare [6]. Indian women were not accorded senior positions, which were only reserved for European women in the hospital. She wrote a public letter about this to the local newspaper. Her commitment to medicine and her desire to provide quality healthcare to women were evident in her work at the hospital. She faced numerous challenges, including discrimination regarding both gender and race. This made her realize that in order to get a respectable position, she needed to pursue medicine abroad.

Dr. Ganguly traveled to the United Kingdom in 1893 to further her medical education. She obtained additional qualifications, including the Licentiate of the Royal College of Physicians (LRCP) in Edinburgh, the Licentiate of the Royal College of Surgeons (LRCS) in Glasgow, and the Graduate of the Faculty of Physicians and Surgeons (GFPS) in Dublin, the very same year [7].

Upon her return to India, Dr. Ganguly focused on her private practice, primarily on gynecology and women's health. Her medical expertise and compassionate care made her a highly sought-after physician, earning her widespread recognition and praise among her peers and patients. Her patients included members of the Nepalese Royal Family and upper-class families in Calcutta [8]. She made significant contributions to the field of women's healthcare, addressing medical issues that had traditionally been overlooked due to social taboos surrounding women's health.

Social reform and activism

Beyond her groundbreaking medical career, Dr. Ganguly was a passionate advocate for social reform. She actively participated in the Brahmo Samaj, a movement dedicated to modernizing Hindu society by abolishing practices like child marriage, dowry, and the oppression of widows while promoting women's education and rights. Ganguly's involvement in this movement exemplified her unwavering commitment to social justice and gender equality. She was a member of the Bengal Ladies’ Association or the Banga Mahila Samaj, which was particularly involved in issues regarding the upliftment of women and their cultural and spiritual well-being.

Furthermore, Dr. Ganguly championed the cause of women's education, recognizing its crucial role in empowering women and improving their socio-economic status. She worked tirelessly to promote access to education for girls, firmly believing that educated women would be better equipped to contribute to society and make informed choices about their lives.

In addition to her work in education, Ganguly was a vocal advocate for the abolition of child marriage, a practice she recognized as detrimental to young girls, posing health risks and depriving them of their childhood and educational opportunities. She was consulted when the British considered raising the age of consent for sexual intercourse from 10 to 12 years. Based on her considerations, the Age of Consent Act, 1891 was passed by the British Government, and the age of cohabitation for women was raised to 12 years, which was a great feat, considering child marriage was a norm in Indian society [9]. Her efforts to raise awareness and advocate for legal reforms were instrumental in catalyzing a shift in societal attitudes toward child marriage. She worked as a member of a committee reporting on the lives of female workers in the coal mines of Bihar and Orissa [10].

Dr. Ganguly was also involved in the Indian National Congress, which was at the forefront of the Indian Independence movement. She was one of the first six women delegates of the fifth session of the Indian National Congress held in 1889. She was the first woman speaker at the sixth session of the Indian National Congress held in 1890 [11]. She was made the first chair of the Transvaal Indian Association in 1907 [12]. Her involvement in the freedom movement demonstrated the convergence of her professional and social activism. She was convinced that achieving political independence and addressing social inequality were closely intertwined, and she dedicated herself to building a society that was liberated from both colonial domination and societal inequities. As a prominent advocate for independence, she acknowledged the essential connection between self-governance and the establishment of a fairer social structure.

Challenges and overcoming barriers

Dr. Ganguly encountered numerous obstacles throughout her journey. Being among the pioneering female individuals in Indian medicine, she confronted substantial opposition from a society unprepared to embrace women in professional capacities. Overcoming gender bias, doubt, and discrimination from both her male peers and the wider community was no easy feat for Dr. Ganguly. In 1891, "Bangabasi," a journal of the Hindu orthodoxy, described her as a whore. She, along with her husband, Dwarakanath Ganguly, sought legal action against the editor, Mahesh Chandra Pal. He was found to be guilty of his actions and was fined Rs. 100 and sentenced to six months' imprisonment [13].

Balancing professional obligations with personal life presented another considerable challenge for Dr. Ganguly. Raising six children along with two stepchildren while pursuing a medical career and engaging in social activism demanded exceptional multitasking abilities, a true reflection of her resilience and unwavering determination.

Furthering her education also posed challenges for Dr. Ganguly as she ventured to the United Kingdom to acquire additional medical qualifications in an era where such endeavors were daunting for women in late 19th-century India. However, her resolve to excel in her profession and her dedication to providing high-quality patient care spurred her to overcome these barriers and reach her objectives.

## Conclusions

Ganguly shattered several glass ceilings, not just for women in medicine but also for women in general. She passed away on October 3, 1923, due to a stroke, but her contributions to medicine and social justice continue to affect current generations alike. Her family's support, including her father Brajakishore Basu and later her husband Dwarkanath Ganguly, who acted as her teacher, companion, and guide, was crucial and indispensable. These individuals played a remarkable role in shaping her life. Her life's work opened doors for future generations of female doctors in India and still inspires both social reformers and medical professionals. Dr. Ganguly's story is a powerful reminder of the importance of perseverance and determination in achieving one’s goals despite facing adversities. As we reflect on her life and legacy, we are reminded of the importance of continuing the work she started, striving for a society that is just, equitable, and inclusive for all.
